# Prevalence of transfusion transmissible infections in blood donors of Pakistan

**DOI:** 10.1186/s12878-016-0068-2

**Published:** 2016-11-18

**Authors:** Aisha Arshad, Munira Borhany, Nida Anwar, Imran Naseer, Rehan Ansari, Samson Boota, Naveena Fatima, Mustansir Zaidi, Tahir Shamsi

**Affiliations:** Department of Blood Bank of National Institute of Blood Disease and Bone Marrow Transplantation (NIBD), St 2/A block 17 Gulshan-e-Iqbal KDA scheme 24, Karachi, Pakistan

**Keywords:** Blood bank, Hepatitis, Syphilis, Pakistan

## Abstract

**Background:**

Transfusion-transmitted infections threaten the safety of patients requiring blood transfusion, which in turn imposes serious challenges for the availability of safe blood products that are still affordable in health care systems with limited resources. The aim of the study was to determine the prevalence of transfusion-transmitted infections in blood donors and to evaluate the demographic characteristics of reactive and non-reactive blood donors.

**Methods:**

A prospective cohort study was conducted at our institute in Karachi, Pakistan. Donors were required to fill a detailed questionnaire and were screened for Hepatitis B, Hepatitis C, Human immunodeficiency viruses, Syphilis and Malaria by ELISA and thick film (malaria).

**Results:**

Of the 16,602 blood donors, 16,557 were males and 45 females (mean age 28.6 ± 2). Nine hundred and seventy three (5.8%) donations were reactive in any screening assay, with 58 (0.35%) donations reacting in more than one assay. The prevalence of Hepatitis B, Hepatitis C, Human immunodeficiency viruses, Syphilis and Malaria was found to be 1.84, 1.7, 0.04, 2.1 and 0.07% respectively. Characteristics among the infections were evaluated and it was found that unmarried donors had a higher chance to be infected by Hepatitis B virus and Syphilis as compared to the other infections. On the other hand, construction workers and married donors were at more risk to be infected by Syphilis rather than the other infections. In case of co-infections, personnel with different occupations and marital status were infected by more than one pathogen.

**Conclusion:**

A substantial percentage of the blood donor’s harbored transfusion-transmitted infections. Prevention of TTIs should be the main goal right now. There is a need for stringent selection of blood donors with the emphasis on getting voluntary donations and comprehensive screening of donor’s blood for TTIs using standard methods to ensure the safety of blood recipient.

## Background

Blood donation saves the lives of millions of people worldwide; however, the patients are at a potential risk of contracting transfusion-transmitted infections (TTIs), which in turn impose serious challenges to the medical providers for the availability of safe and affordable blood products. According to the World health organization (WHO), safe blood is a universal right. A crucial requirement in the procurement of safe blood is to have a national program for donor selection, recruitment, retention, and education; this will minimize donations from donors who might transmit diseases to the recipients. Equally important is to evaluate the burden and risk factors for TTIs in the general population [1]. The accurate figures of TTIs in our population are still unknown due to the lack of understanding, un-availability of screening tests, limited access to health facilities and the unavailability of surveillance systems [2]. Furthermore, voluntary donors have been reported to be the safest group of donors because they usually have better health seeking behavior than the replacement blood donors and their intention is to donate blood to an unknown patient out of compassion [3].

In Pakistan, more than 1.5 million units of blood are collected each year but the majority of these donors are replacement donors, mostly family members or close friends of the patient with the intention to help in most cases for transfusion under emergency situations [4]. There is an immense need to provide safe blood products. This requires high quality transfusion services and an organized infrastructure along with properly trained and well-educated staff [2].

The prevalence of Hepatitis B virus (HBV) and Hepatitis C virus (HCV) in Pakistan is high and has been reported earlier (HBV: 2.5% and HCV: 4.9%) [4]. However recent studies raised concerns regarding the increasing prevalence in trends of TTIs other than HBV and HCV, especially Human immunodeficiency viruses (HIV) and Syphilis [2, 3, 5–8]. The present study provides data on the overall seroprevalence of TTIs in blood donors and evaluates the demographic characteristics of seropositive donors.

## Methods

This was a prospective cohort study of blood donors attending the National Institute of Blood disease and Bone Marrow transplantation (NIBD) donor’s center from January 2013 to June 2015. Informed consent was taken from all donors. Blood donors were given a unique identification number and their name, age, sex, date of birth, profession, marital status and contact numbers were recorded. Before the donation, each potential donor was required to fill a detailed health history questionnaire which included data regarding their general health, life style, current or past febrile illness, weight loss, chronic disease, unusual or excessive bleeding, drug history, tattoo piercing, dental treatment, previous blood donation or transfusion, history of travel or immigration, sexual history and risk behaviors followed by short private interviews. Vitals and weight were also recorded. Baseline complete blood count (CBC) was done for excluding any donors with anemia (<12.5 g/dl), infection or thrombocytopenia. Moreover, inspection was made for any marks of drug abuse or any skin lesion at the veni puncture site. Proper sterilization and other precautions were taken during blood collection and blood units were stored using appropriate methods. Blood donors were then screened for HBV, HCV, HIV, Syphilis and Malaria.

### Laboratory tests

Screening for HBsAg, Anti HCV, HIV Ag/Ab (HIV-1/HIV-2) and Syphilis was done by chemiluminescent micro particle immunoassay (CMIA) method on Architect i2000 (Abbott Diagnostic, USA). Furthermore, Malaria was screened by thick films and through the immunochromatographic test (ICT) method. Reactive results were repeated on the same sample using the same method.

### Study subjects inclusion criteria

Physically fit 18–55 year olds who donated blood at NIBD blood donor center were included.

### Study subjects exclusion criteria

Potential donors were excluded if they were: below 18 years old, weigh <50 kg, anemic, had a history of jaundice, malaria, asthma, engaged in high risk behavior (i.e. unsafe intercourse, drug abuse), had past history of HBV, HCV, HIV I & II or syphilis, or were apparently unhealthy or malnourished.

### Statistical analysis

Data was analyzed using SPSS version 21. Infections among reactive and nonreactive blood donors were analyzed by using logistic regression and estimates of the odds ratio with their corresponding 95% confidence interval. Chi Square was applied to find the association of co-infections with demographic variables. *P*-value <0.05 was considered significant in all analysis.

## Results

A total of 16,602 donors who visited for blood donation were screened; 16,557 were males and 45 females, with a mean age of 28.6 ± 2 years (range 18 to 55 years), of which 95% were replacement blood donors. Of all donations, 973 (5.8%) were reactive in the screening assays, among them 58 (0.35%) were reactive in more than one assay. The prevalence of HCV, HBV, HIV, syphilis and malaria in our study population was 1.8, 1.7, 0.04, 2.1 and 0.07% respectively. The overall demographic variables among the reactive and non-reactive donors are shown in (Table 1). Characteristics among infections were individually evaluated, as shown in (Table 2). It was found that unmarried donors were more likely to be positive for HBV infection (OR-CI: 0.331:0.202-0.570, *P*-value: 0.001) and Syphilis (OR-CI: 0.182:0.065-0.512, *P*-value: 0.001) rather than the other infections. The construction workers (OR-CI: 0.219:0.129-0.370, *P*-value: 0.000) and married persons (OR-CI: 0.360:0.158-0.820, *P*-value: 0.015) have a higher chance to be positive for Syphilis than the other infections. The association of gender, age groups, education, number of donations, and residential status was not significant for any of the studied infections. We observed the trend of co–infections (risk to be infected by more than one pathogens) in the sub categories of our donors and it was found that the subcategory of occupation (*P*-value = 0.01) and the subcategory of marital status (*P*-value = 0.00) were significant. (Table 3)

**Table 1:** Demographic variables of reactive and non-reactive blood donors

Characteristics		
	Non-React.	React.
Age groups		
18–25	8010	466
26–35	6110	402
More than 35	1509	105
Gender		
Female	42	3
Male	15587	970
Occupation		
Student	1471	110
Businessman	4000	173
Driver	3790	269
Employee	2789	197
Construction worker/laborer	4190	224
Education		
Illiterate	6420	180
Literate	9209	793
Number of donations		
Repeat donation	4774	128
First donation	10855	845
Marital status		
Divorced	1268	742
Unmarried	8864	143
Married	5497	88
Resident		
Rural	5863	110
Urban	9766	863

**Table 2:** Demographic variables of individual Infections among reactive and non-reactive blood donors

Characteristics	HBsAg (*n*=290)			Anti HCV (*n*=307)			MP (*n*=12)			Syphilis (*n*=357)		
	Non react.	React	*P*-value	Non Ract.	React	*P*-value	Not seen	Seen	*P*-value	Non react.	React	*P*-value
Age groups												
18–25	8314	156	0.847	8340	131	0.616	8468	7	0.987	8291	170	0.177
26–35	6394	111	0.745	6364	144	0.063	6501	5	0.988	6357	154	0.357
More than 35*	1604	23		1591	32		1629	0		1597	33	0.861
Gender												
Female	45	0	0.998	44	1	0.504	45	0	0.998	45	0	0.998
Male*	16267	290		16251	306		16541	12		16186	357	
Occupation												
Student*	1508	54		2496	54		1770	0		2077	36	
Businessman	4076	77	0.924	3796	77	0.986	3182	5	0.999	3727	28	0.220
Driver	3776	52	0.977	3596	61	0.997	4753	3	0.997	3486	50	0.593
Office Employee	2776	32	0.982	2848	41	0.996	3640	0	0.97	3736	22	0.989
Construction worker/laborer	4176	75	0.977	3559	74	0.997	3245	4	0.995	3219	221	0.000
Education												
Illiterate*	6408	169	0.975	5242	178	0.972	6185	6	0.095	5192	233	0.686
Literate	9904	121		11053	129		10405	6		11053	124	
Number of donations												
Repeat donation*	4783	106	0.986	4506	134	0.984	6203	2	0.936	4419	128	0.053
First donation	11529	184		11789	173		10387	10		11826	229	
Marital status												
Divorced*	2259	74		1480	81		4873	1		1513	13	
Unmarried	8327	136	0.00	7765	146	0.985	7911	5	0.959	7837	83	0.001
Married	5726	80	0.987	7050	80	0.979	3806	6	0.938	6895	261	0.015
Residential status												
Rural	5852	103	0.994	7748	158	0.999	6802	6	0.948	7670	267	0.826
Urban*	10460	187		8547	149		9788	6		8575	90	

*reference category

**Table 3:** Association of Co-infections with demographic variables

Characteristics	Co-Infections (*N* = 58)		
	Non reactive	Reactive	*P*-value
Age groups			
18–25	8446	30 (0.35%)	0.791
26–35	6491	21 (0.32%)	
More than 35	1607	7 (0.43%)	
Gender			
Female	45	0	0.691
Male	16,499	58 (0.35%)	
Occupation			
Student	1433	4 (0.27%)	0.01
Businessman	4080	6 (0.14%)	
Driver	3755	23 (0.61%)	
Office employee	2735	12 (0.43%)	
Construction worker/laborer	4177	13 (0.31%)	
Education			
Illiterate	6597	29 (0.43%)	0.11
Literate	9947	29 (0.29%)	
Number of donations			
Repeat donation	4899	17 (0.34%)	0.95
First donation	11,624	41 (0.35%)	
Marital status			
Divorced	1954	19 (0.97%)	0.00
Unmarried	8141	22 (0.27%)	
Married	5585	17 (0.30%)	
Residential status			
Rural	5974	28 (0.46%)	0.54
Urban	10,570	30 (0.28%)	

## Discussion

Blood transfusion is a life-saving procedure of modern medicine. Stringent screening of blood not only gives us an idea about the prevalence of TTIs in healthy populations but also ensures the safe supply of blood and blood products. [9]. Disease burden estimations based on sound epidemiological research form the basis of public policy. Similarly, the exact evaluations of the risk of TTIs is imperative in order to monitor the safety of blood supply and gauging the effectiveness of the presently employed screening procedures, as discussed by Busch et al. [10]. According to one study, in Pakistan the majority of the blood donors are first timers, which can be considered a true reflection of infection amongst the community [9]. However, according to other studies, blood donors may not represent the general population as the prevalence rate may be underestimated or overestimated due to their different characteristics [11, 12]. Since most of them are male, young or middle-aged, the source of prevalence may underestimate the actual prevalence. This view seems more valid as opposed to the previous one simply because females comprise more than 50% of Pakistan’s population. The prevalence of TTIs among blood donors in a well-structured health care system coupled with a well-organized blood establishment can be used as a reliable tool for statistical calculation of those infectious agents that can be transmitted through blood products in the populations, as discussed by Gharehbaghian and Chandra et al. [1, 13].

In our study, most of the donors were replacement blood donors i.e. 95% which is comparable with other local studies in which majority of blood donations were contributed by replacement donors [4, 14] with the intention to help a friend, relative or acquaintance who needed blood transfusion. The maximum number of donors came from the 18–30 year age group. A similar trend was seen in earlier reports [15, 16]. Furthermore, efforts should also be made to encourage and improve the number of female donors, as our study shows limited number of female donors. In this study, a significant increase in the seroprevalence of syphilis was observed among blood donors over the study period, which was found to be 2.1%. A local study that was done previously had also observed a rising seroprevalence of 0.89% [17]. This finding is consistent with the increasing trend of syphilis seropositivity observed in blood donors of Israel [18]. A study done in Iran also found a rising trend of syphilis frequency in their population; however, the frequency found in this study was 0.04% [19]. Previous local data shows low seroprevalence from 0.22 to 0.89%, which is contrary to that observed in our study [4, 20–22]. Another study found a downward trend in seroprevalence [3]. Moreover, Moiz et al. observed similar results in their study [23]. Furthermore, studies done in India had very low prevalence as compared to our study [15, 24]. Thus, the seroprevalence of syphilis in blood donors observed in our study was high as compared to the previous local and international data. Since citizens perform blood donation(s), it could provide an updated picture about prevalence of Syphilis in the Pakistani population.

However, one of the limitations in our study is that positivity of Syphilis could not be further validated by confirmatory assay and positivity could only give indication as a surrogate marker.

There is also limited surveillance data for HIV in Pakistan. Current study reports the figure of 0.04%, which is slightly higher than the one reported in studies by Attaullah, Manzoor and Sultan et al. [2, 4, 21]. HIV positive blood units were confirmed with other techniques in the reference HIV laboratory.

Acute and chronic viral hepatitis are the most reported health problems in Pakistan and usually bring with them serious complications. Local data regarding the prevalence of HBV and HCV infections among healthy blood donors is well recognized [12, 14, 17, 25] except by the Mahmood et al. study [26], which shows less percentage of HBsAg and anti HCV.

Risk behavior–based donor selection is the cornerstone of the availability of safe blood. It is dependent on donor education as well as the accurate and truthful disclosure of risk behavior [12]. In our study, risk factors were assessed using a questionnaire but many blood donors were reluctant to disclose any risk behavior(s) because of the potential fear of judgment, embarrassment, test-seeking behavior, or due the genuine belief that their blood was safe [12]. We have over all observed risk factors among the blood donors in our study however they were not analyzed for each blood donor’s category individually. Therefore, we could only simulate risk factors among blood donors based on local studies on HBV and HCV infections [11, 12].

The focus of our study was also to determine the demographic characteristics of reactive blood donors. Positive donors for any infection were informed and requested to visit general physicians. A short interview was conducted to find more details regarding the life styles of reactive blood donors. This is the first local study in our region, which shows detailed demographic characteristics information regarding blood donors.

## Conclusion

A substantial percentage of the blood donor’s harbored transfusion transmitted infections. Prevention of TTIs should be the main goal right now. There is a need for stringent selection of blood donors with the emphasis on getting voluntary donations and comprehensive screening of donor’s blood for HCV, HBV, HIV, Syphilis and Malaria using standard methods to safeguard the blood recipient. By assessing the rising trend of Syphilis in Pakistan, special attention should be given to targeted blood donor’s population and the population at risk. A future study is also planned to assess the knowledge, attitude and behavior of the blood donor’s population. These measures will improve public health and would increase blood safety and quality.

## Abbreviations

CBC: Complete blood count
CMIA: Chemiluminescent micro particle immunoassay
HBV: Hepatitis B virus
HCV: Hepatitis C virus
HIV: human immunodeficiency virus
ICT: Immunochromatographic test
TTIs: Transfusion-transmitted infections
WHO: World Health Organization
